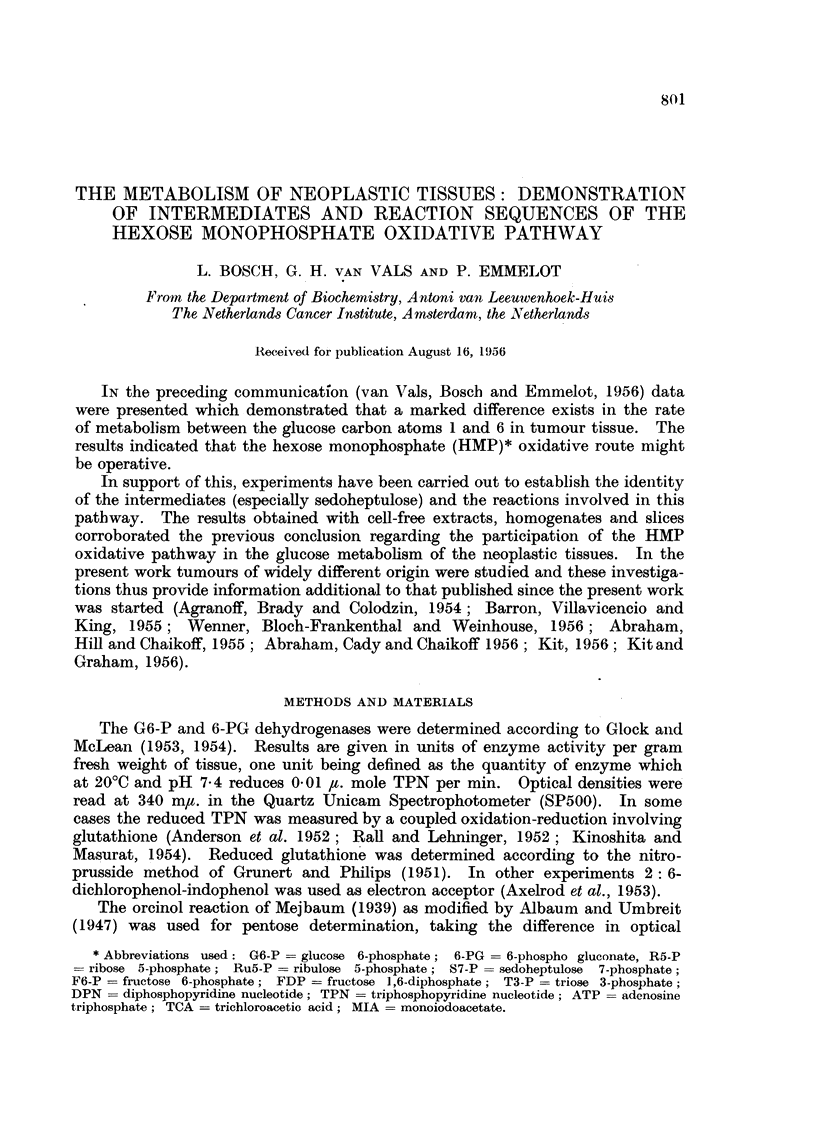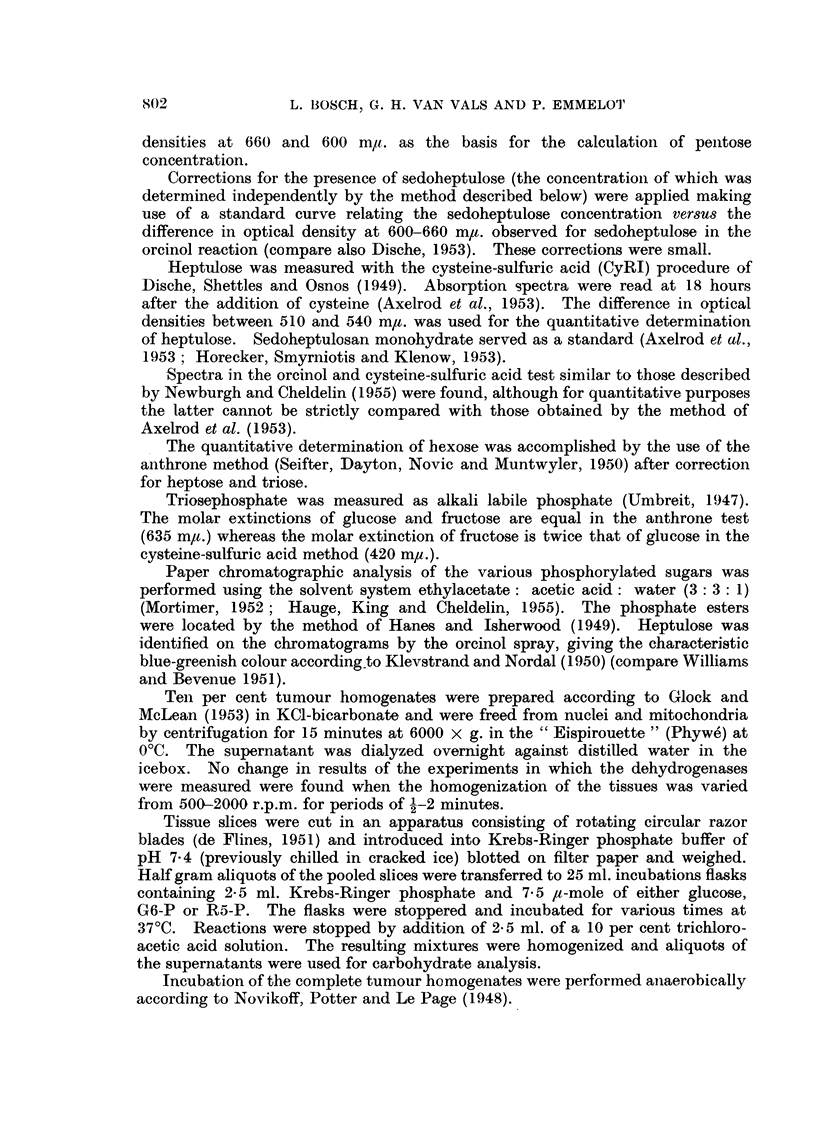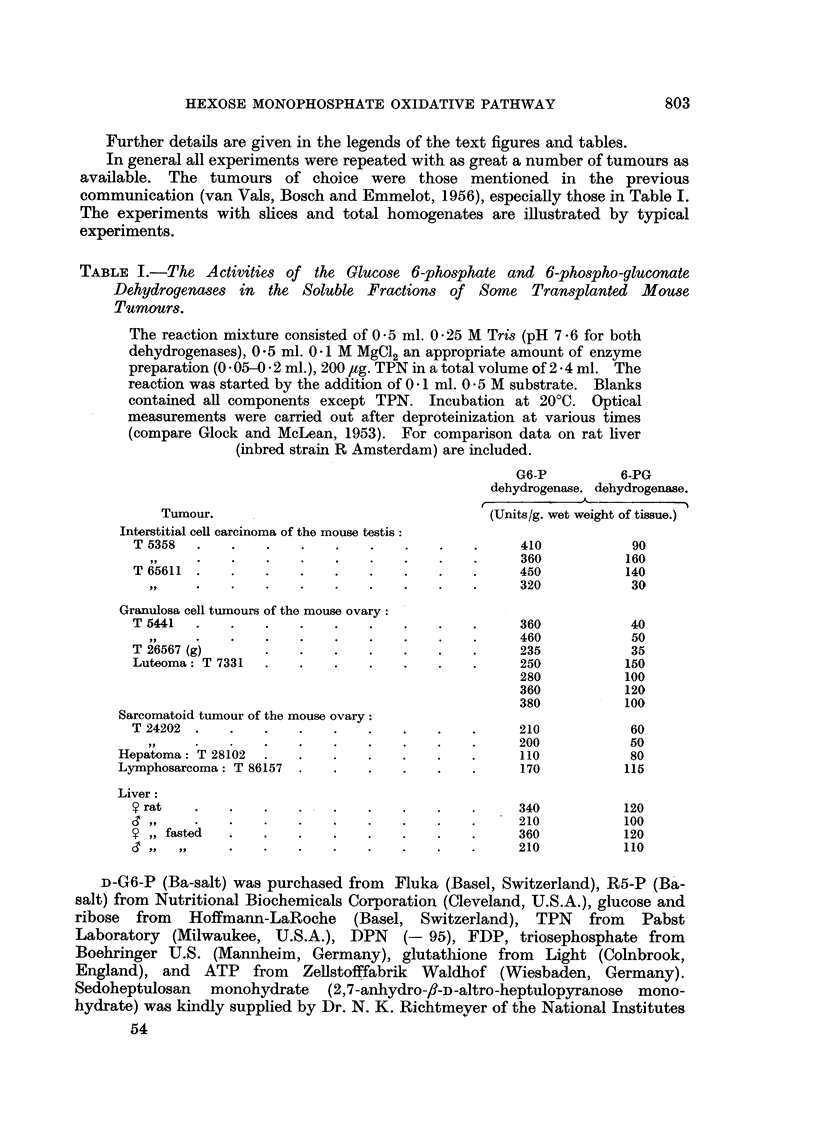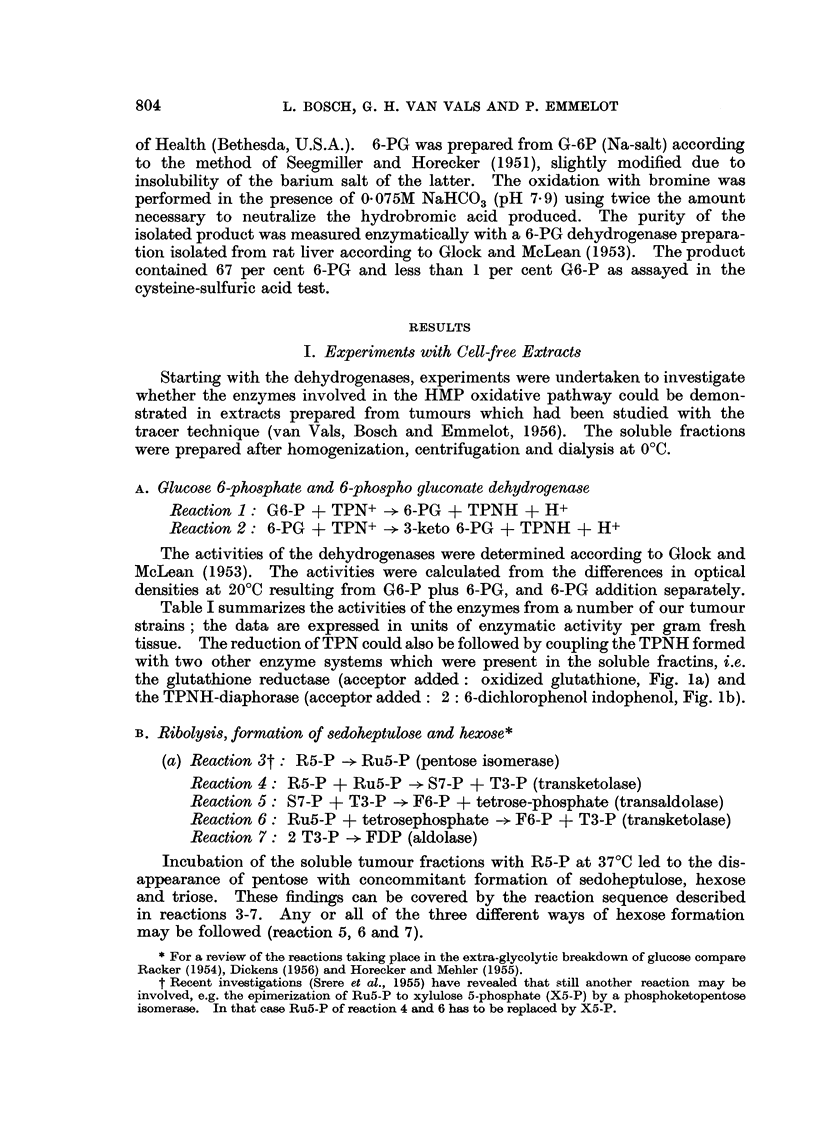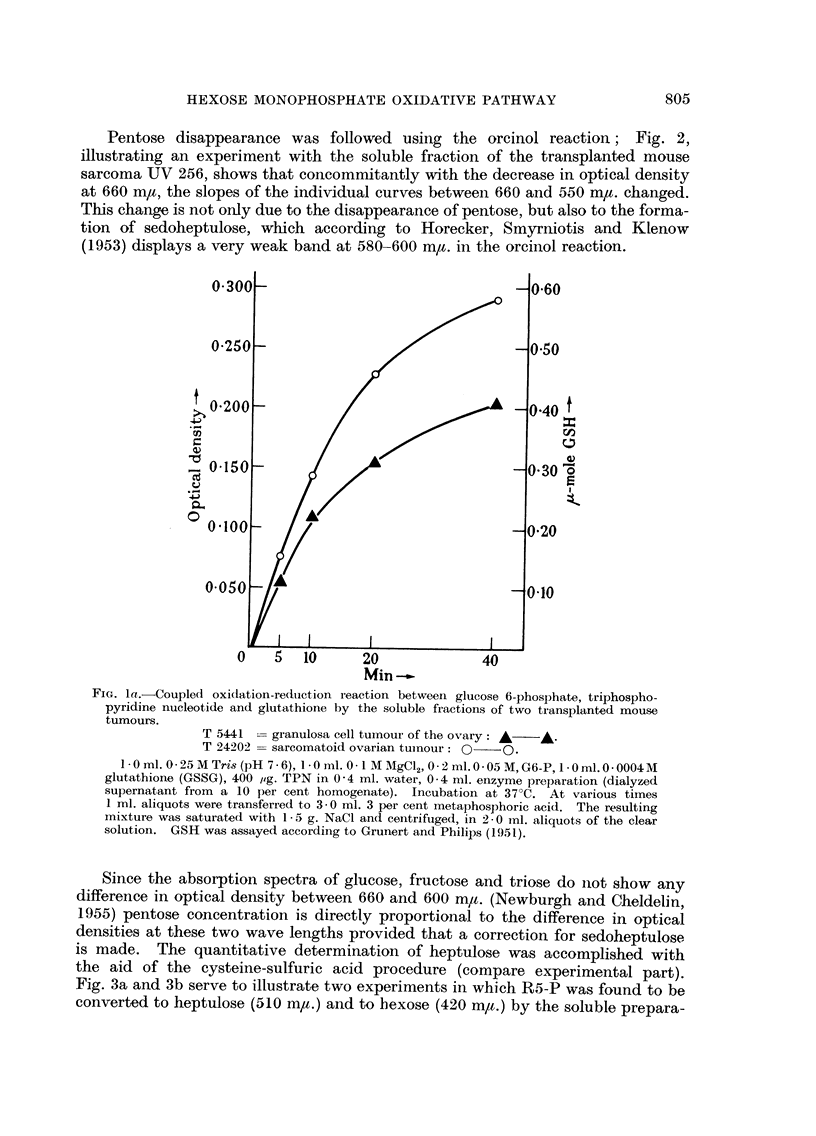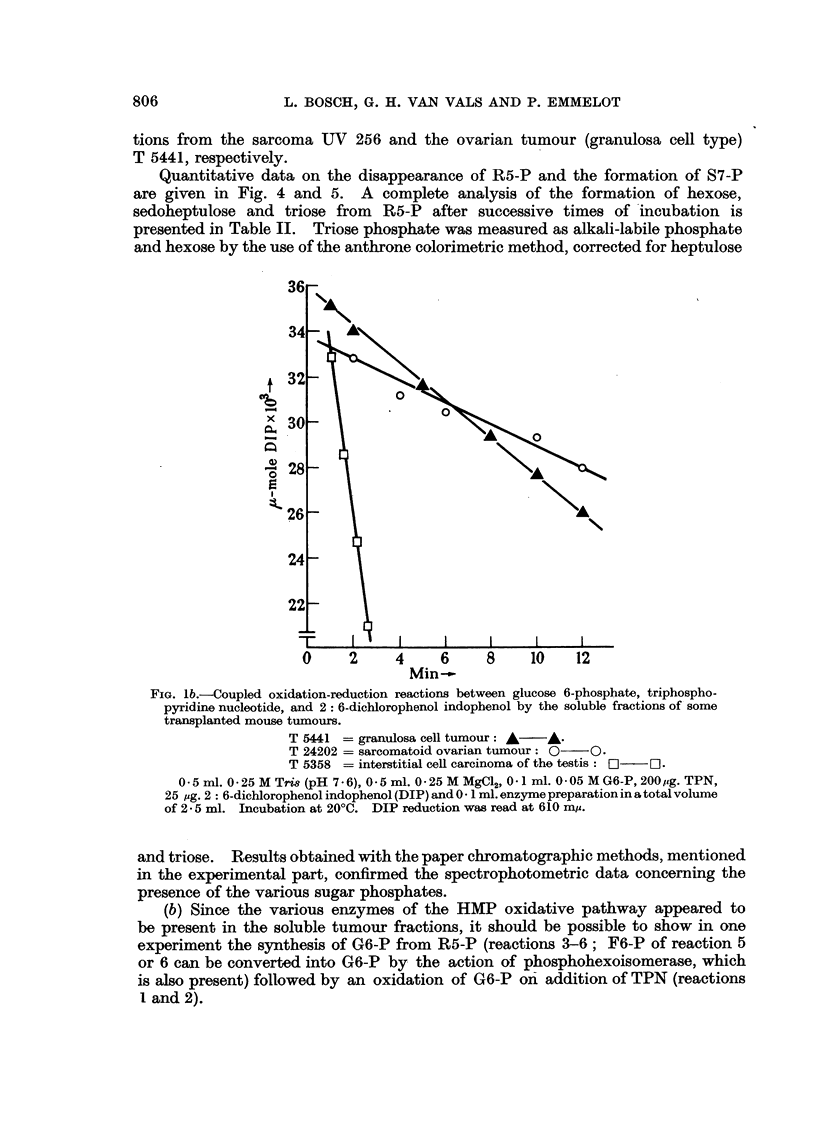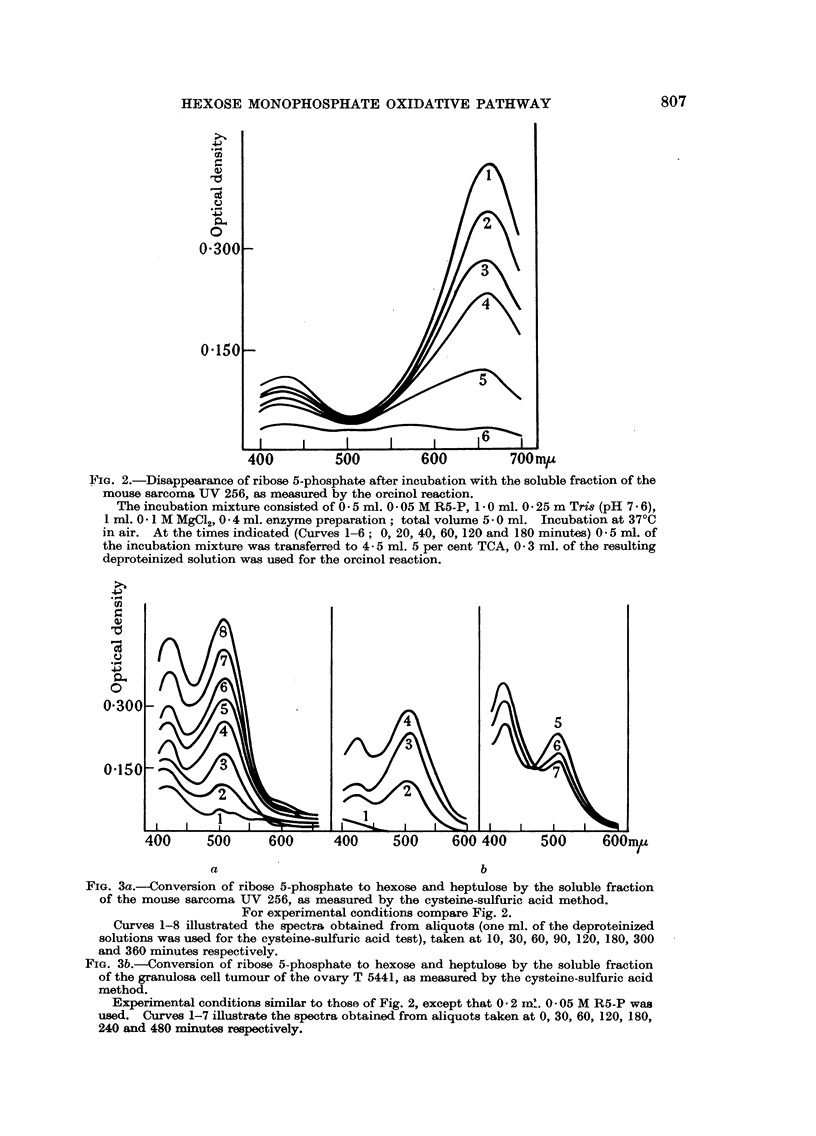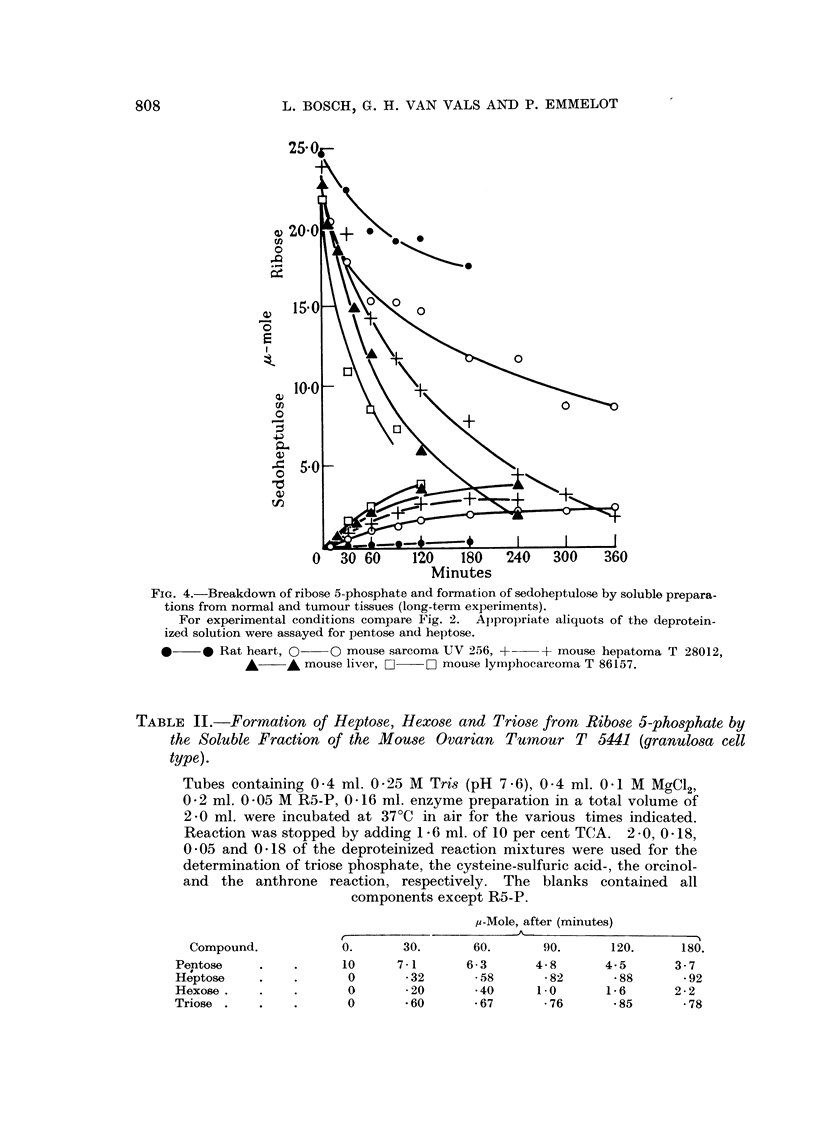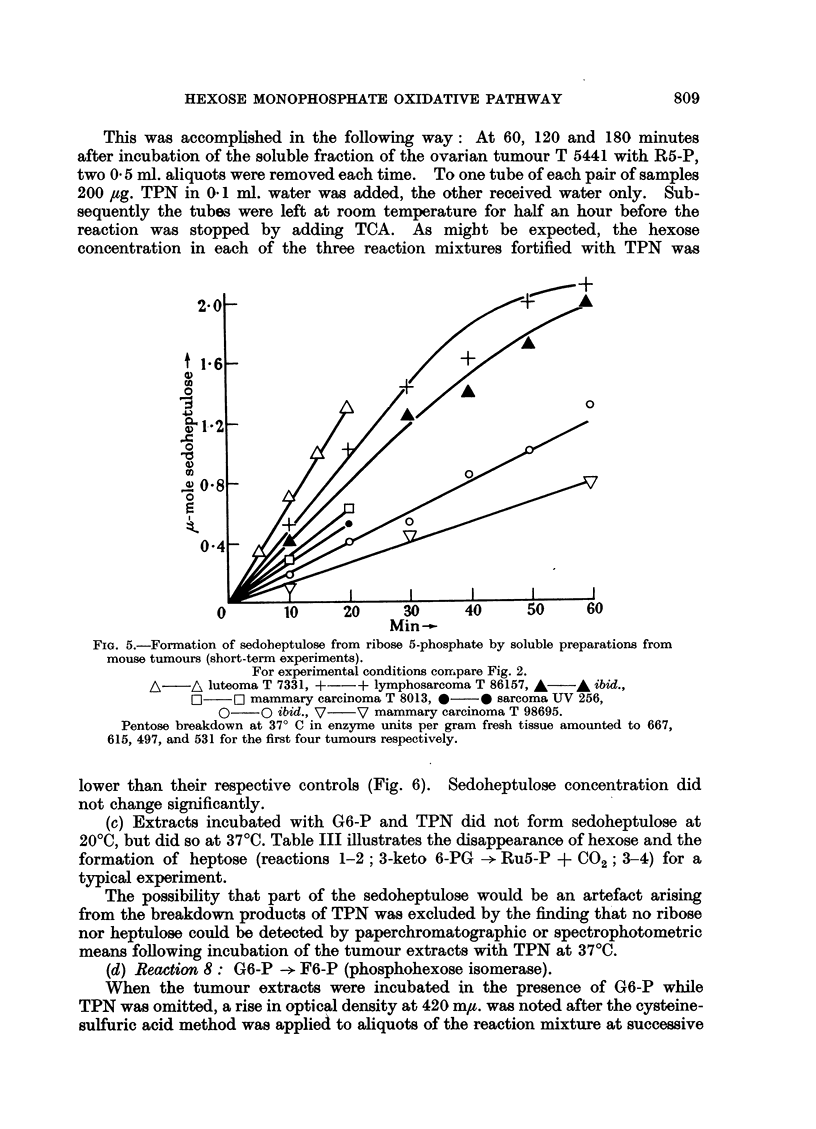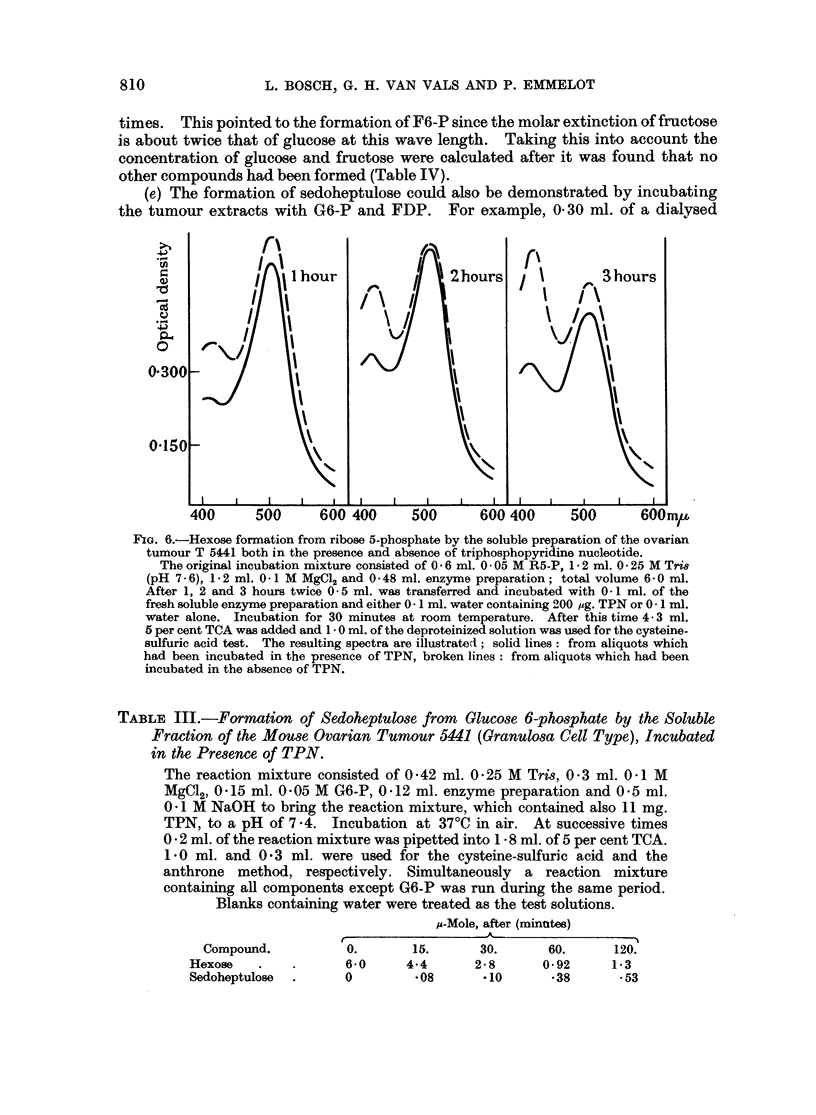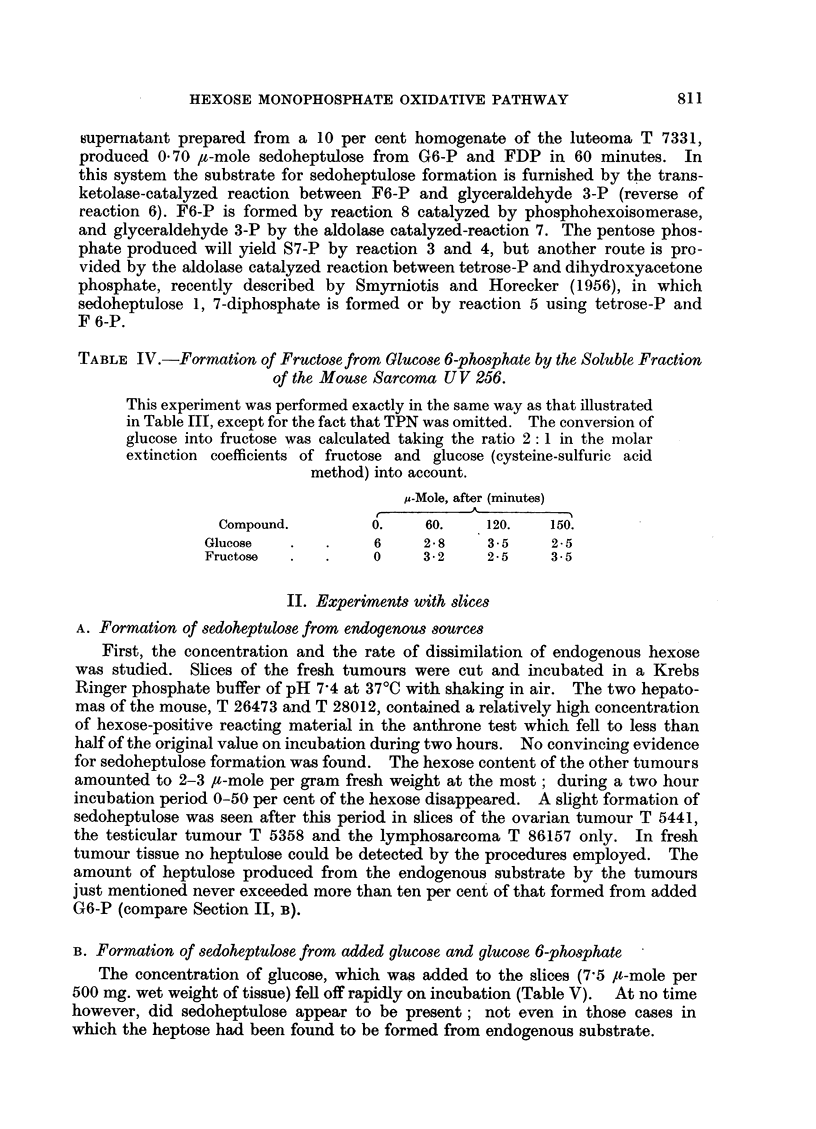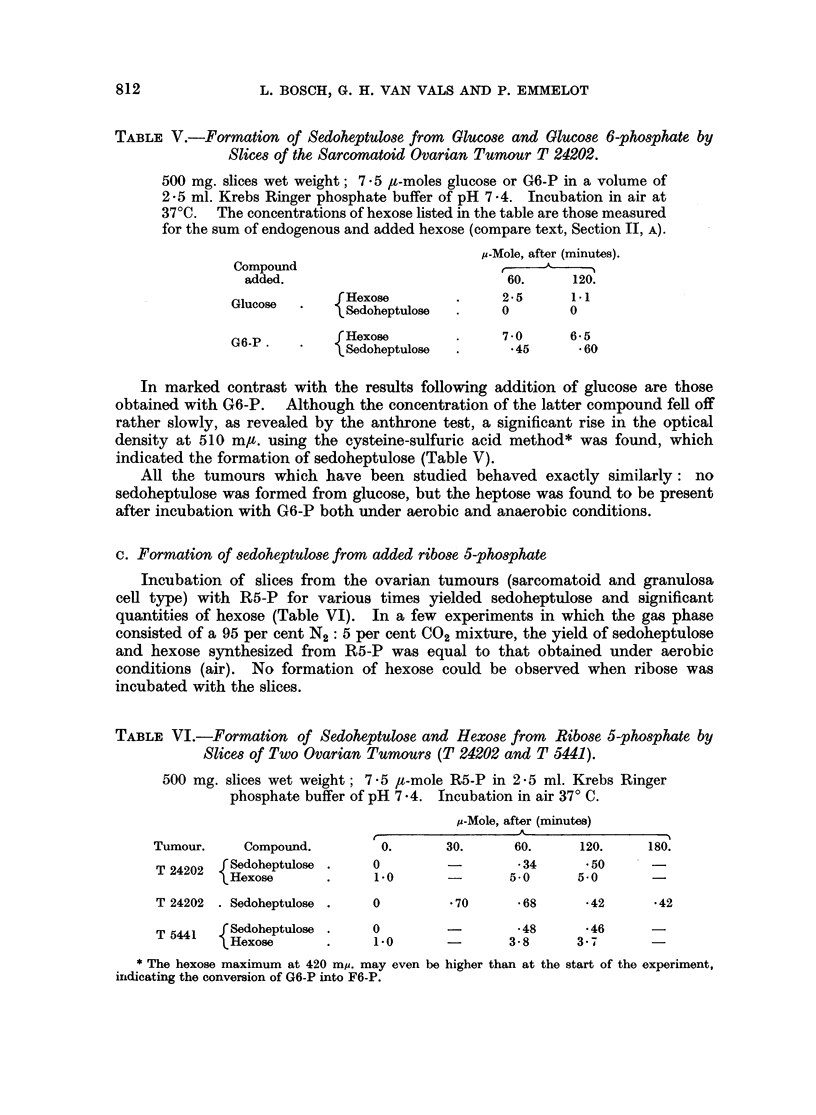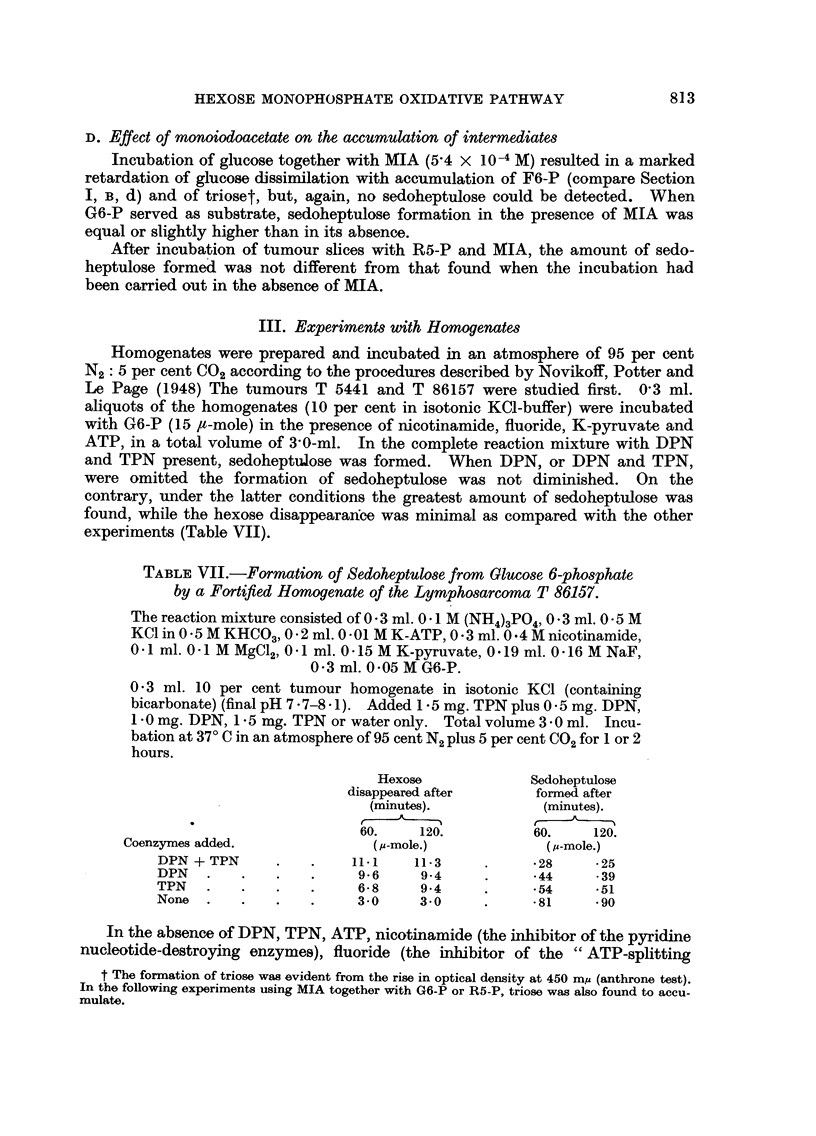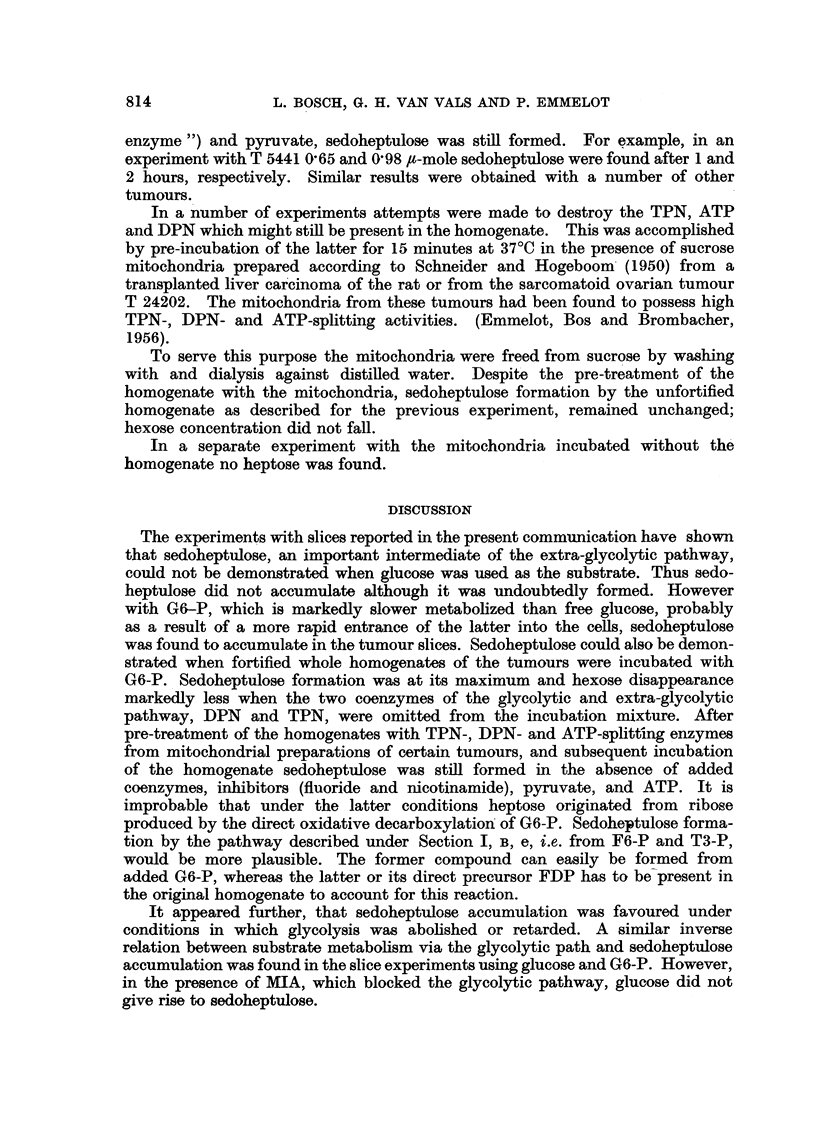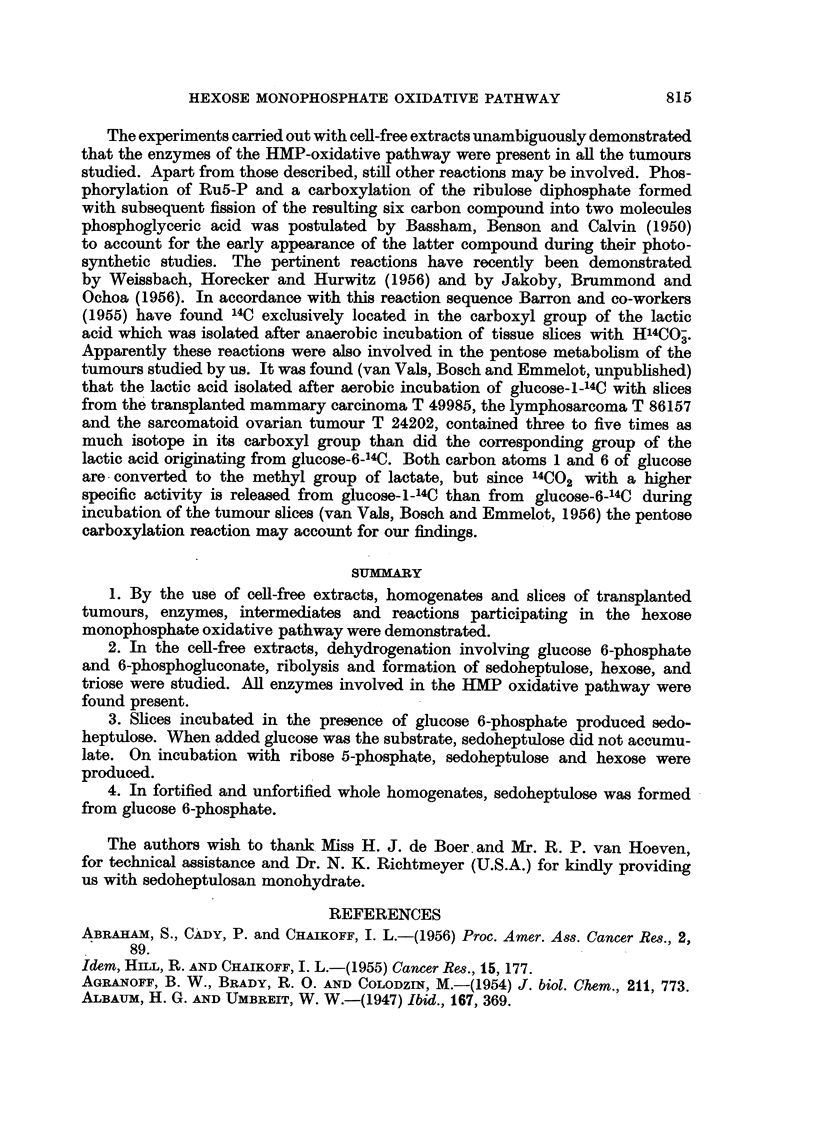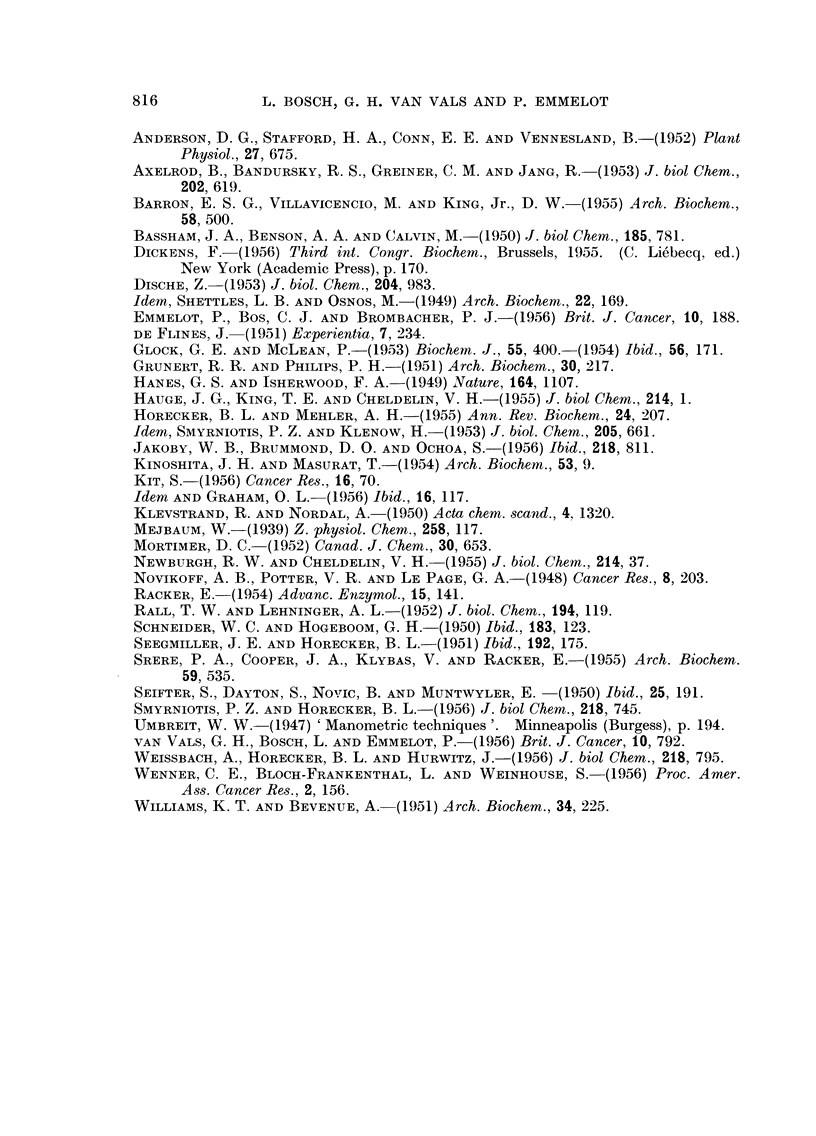# The Metabolism of Neoplastic Tissues: Demonstration of Intermediates and Reaction Sequences of the Hexose Monophosphate Oxidative Pathway

**DOI:** 10.1038/bjc.1956.97

**Published:** 1956-12

**Authors:** L. Bosch, G. H. van Vals, P. Emmelot


### THE METABOLISM OF NEOPLASTIC TISSUES: DEMONSTRATION OF INTERMEDIATES AND REACTION SEQUENCES OF THE HEXOSE MONOPHOSPHATE OXIDATIVE PATHWAY

L. BOSCH, G. H. VAN VALS AND P. EMMELOT

*From the Department of Biochemistry, Antoni van Leeuwenhoek-Huis The Netherlands Cancer Institute, Amsterdam, the Netherlands*

Received for publication August 16, 1956

IN the preceding communication (van Vals, Bosch and Emmelot, 1956) data were presented which demonstrated that a marked difference exists in the rate of metabolism between the glucose carbon atoms 1 and 6 in tumour tissue. The results indicated that the hexose monophosphate (HMP)* oxidative route might be operative.

In support of this, experiments have been carried out to establish the identity of the intermediates (especially sedoheptulose) and the reactions involved in this pathway. The results obtained with cell-free extracts, homogenates and slices corroborated the previous conclusion regarding the participation of the HMP oxidative pathway in the glucose metabolism of the neoplastic tissues. In the present work tumours of widely different origin were studied and these investigations thus provide information additional to that published since the present work was started (Agranoff, Brady and Colodzin, 1954; Barron, Villavicencio and King, 1955; Wenner, Bloch-Frankenthal and Weinhouse, 1956; Abraham, Hill and Chaikoff, 1955; Abraham, Cady and Chaikoff 1956; Kit, 1956; Kit and Graham, 1956).

### METHODS AND MATERIALS

The G6-P and 6-PG dehydrogenases were determined according to Glock and McLean (1953, 1954). Results are given in units of enzyme activity per gram fresh weight of tissue, one unit being defined as the quantity of enzyme which at 20°C and pH 7·4 reduces 0·01 $\mu$. mole TPN per min. Optical densities were read at 340 m$\mu$. in the Quartz Unicam Spectrophotometer (SP500). In some cases the reduced TPN was measured by a coupled oxidation-reduction involving glutathione (Anderson *et al.* 1952; Rall and Lehninger, 1952; Kinoshita and Masurat, 1954). Reduced glutathione was determined according to the nitroprusside method of Grunert and Philips (1951). In other experiments 2:6-dichlorophenol-indophenol was used as electron acceptor (Axelrod *et al.*, 1953).

The orcinol reaction of Mejbaum (1939) as modified by Albaum and Umbreit (1947) was used for pentose determination, taking the difference in optical

* Abbreviations used: G6-P = glucose 6-phosphate; 6-PG = 6-phospho gluconate, R5-P = ribose 5-phosphate; Ru5-P = ribulose 5-phosphate; S7-P = sedoheptulose 7-phosphate; F6-P = fructose 6-phosphate; FDP = fructose 1,6-diphosphate; T3-P = triose 3-phosphate; DPN = diphosphopyridine nucleotide; TPN = triphosphopyridine nucleotide; ATP = adenosine triphosphate; TCA = trichloroacetic acid; MIA = monoiodoacetate.

densities at 660 and 600 mμ. as the basis for the calculation of pentose concentration.

Corrections for the presence of sedoheptulose (the concentration of which was determined independently by the method described below) were applied making use of a standard curve relating the sedoheptulose concentration *versus* the difference in optical density at 600–660 mμ. observed for sedoheptulose in the orcinol reaction (compare also Dische, 1953). These corrections were small.

Heptulose was measured with the cysteine-sulfuric acid (CyRI) procedure of Dische, Shettles and Osnos (1949). Absorption spectra were read at 18 hours after the addition of cysteine (Axelrod *et al.*, 1953). The difference in optical densities between 510 and 540 mμ. was used for the quantitative determination of heptulose. Sedoheptulosan monohydrate served as a standard (Axelrod *et al.*, 1953 ; Horecker, Smyrniotis and Klenow, 1953).

Spectra in the orcinol and cysteine-sulfuric acid test similar to those described by Newburgh and Cheldelin (1955) were found, although for quantitative purposes the latter cannot be strictly compared with those obtained by the method of Axelrod *et al.* (1953).

The quantitative determination of hexose was accomplished by the use of the anthrone method (Seifter, Dayton, Novic and Muntwyler, 1950) after correction for heptose and triose.

Triosephosphate was measured as alkali labile phosphate (Umbreit, 1947). The molar extinctions of glucose and fructose are equal in the anthrone test (635 mμ.) whereas the molar extinction of fructose is twice that of glucose in the cysteine-sulfuric acid method (420 mμ.).

Paper chromatographic analysis of the various phosphorylated sugars was performed using the solvent system ethylacetate : acetic acid : water (3 : 3 : 1) (Mortimer, 1952 ; Hauge, King and Cheldelin, 1955). The phosphate esters were located by the method of Hanes and Isherwood (1949). Heptulose was identified on the chromatograms by the orcinol spray, giving the characteristic blue-greenish colour according to Klevstrand and Nordal (1950) (compare Williams and Bevenue 1951).

Ten per cent tumour homogenates were prepared according to Glock and McLean (1953) in KCl-bicarbonate and were freed from nuclei and mitochondria by centrifugation for 15 minutes at 6000 × g. in the " Eispirouette " (Phywé) at 0°C. The supernatant was dialyzed overnight against distilled water in the icebox. No change in results of the experiments in which the dehydrogenases were measured were found when the homogenization of the tissues was varied from 500–2000 r.p.m. for periods of ½–2 minutes.

Tissue slices were cut in an apparatus consisting of rotating circular razor blades (de Flines, 1951) and introduced into Krebs-Ringer phosphate buffer of pH 7·4 (previously chilled in cracked ice) blotted on filter paper and weighed. Half gram aliquots of the pooled slices were transferred to 25 ml. incubations flasks containing 2·5 ml. Krebs-Ringer phosphate and 7·5 μ-mole of either glucose, G6-P or R5-P. The flasks were stoppered and incubated for various times at 37°C. Reactions were stopped by addition of 2·5 ml. of a 10 per cent trichloroacetic acid solution. The resulting mixtures were homogenized and aliquots of the supernatants were used for carbohydrate analysis.

Incubation of the complete tumour homogenates were performed anaerobically according to Novikoff, Potter and Le Page (1948).

Further details are given in the legends of the text figures and tables.

In general all experiments were repeated with as great a number of tumours as available. The tumours of choice were those mentioned in the previous communication (van Vals, Bosch and Emmelot, 1956), especially those in Table I. The experiments with slices and total homogenates are illustrated by typical experiments.

TABLE I.—*The Activities of the Glucose 6-phosphate and 6-phospho-gluconate Dehydrogenases in the Soluble Fractions of Some Transplanted Mouse Tumours.*

The reaction mixture consisted of 0·5 ml. 0·25 M *Tris* (pH 7·6 for both dehydrogenases), 0·5 ml. 0·1 M $MgCl_2$ an appropriate amount of enzyme preparation (0·05–0·2 ml.), 200 µg. TPN in a total volume of 2·4 ml. The reaction was started by the addition of 0·1 ml. 0·5 M substrate. Blanks contained all components except TPN. Incubation at 20°C. Optical measurements were carried out after deproteinization at various times (compare Glock and McLean, 1953). For comparison data on rat liver (inbred strain R Amsterdam) are included.

| Tumour. | G6-P dehydrogenase. (Units/g. wet weight of tissue.) | 6-PG dehydrogenase. (Units/g. wet weight of tissue.) |
|---|---|---|
| Interstitial cell carcinoma of the mouse testis : | | |
| T 5358 | 410 | 90 |
| ,, | 360 | 160 |
| T 65611 | 450 | 140 |
| ,, | 320 | 30 |
| Granulosa cell tumours of the mouse ovary : | | |
| T 5441 | 360 | 40 |
| ,, | 460 | 50 |
| T 26567 (g) | 235 | 35 |
| Luteoma : T 7331 | 250 | 150 |
| | 280 | 100 |
| | 360 | 120 |
| | 380 | 100 |
| Sarcomatoid tumour of the mouse ovary : | | |
| T 24202 | 210 | 60 |
| ,, | 200 | 50 |
| Hepatoma : T 28102 | 110 | 80 |
| Lymphosarcoma : T 86157 | 170 | 115 |
| Liver : | | |
| ♀ rat | 340 | 120 |
| ♂ ,, | 210 | 100 |
| ♀ ,, fasted | 360 | 120 |
| ♂ ,, ,, | 210 | 110 |

D-G6-P (Ba-salt) was purchased from Fluka (Basel, Switzerland), R5-P (Ba-salt) from Nutritional Biochemicals Corporation (Cleveland, U.S.A.), glucose and ribose from Hoffmann-LaRoche (Basel, Switzerland), TPN from Pabst Laboratory (Milwaukee, U.S.A.), DPN (— 95), FDP, triosephosphate from Boehringer U.S. (Mannheim, Germany), glutathione from Light (Colnbrook, England), and ATP from Zellstofffabrik Waldhof (Wiesbaden, Germany). Sedoheptulosan monohydrate (2,7-anhydro-β-D-altro-heptulopyranose monohydrate) was kindly supplied by Dr. N. K. Richtmeyer of the National Institutes of Health (Bethesda, U.S.A.). 6-PG was prepared from G-6P (Na-salt) according to the method of Seegmiller and Horecker (1951), slightly modified due to insolubility of the barium salt of the latter. The oxidation with bromine was performed in the presence of 0·075M $NaHCO_3$ (pH 7·9) using twice the amount necessary to neutralize the hydrobromic acid produced. The purity of the isolated product was measured enzymatically with a 6-PG dehydrogenase preparation isolated from rat liver according to Glock and McLean (1953). The product contained 67 per cent 6-PG and less than 1 per cent G6-P as assayed in the cysteine-sulfuric acid test.

### RESULTS

### I. *Experiments with Cell-free Extracts*

Starting with the dehydrogenases, experiments were undertaken to investigate whether the enzymes involved in the HMP oxidative pathway could be demonstrated in extracts prepared from tumours which had been studied with the tracer technique (van Vals, Bosch and Emmelot, 1956). The soluble fractions were prepared after homogenization, centrifugation and dialysis at 0°C.

A. *Glucose 6-phosphate and 6-phospho gluconate dehydrogenase*

*Reaction 1 :* G6-P + $TPN^+$ → 6-PG + TPNH + $H^+$
*Reaction 2 :* 6-PG + $TPN^+$ → 3-keto 6-PG + TPNH + $H^+$

The activities of the dehydrogenases were determined according to Glock and McLean (1953). The activities were calculated from the differences in optical densities at 20°C resulting from G6-P plus 6-PG, and 6-PG addition separately.

Table I summarizes the activities of the enzymes from a number of our tumour strains ; the data are expressed in units of enzymatic activity per gram fresh tissue. The reduction of TPN could also be followed by coupling the TPNH formed with two other enzyme systems which were present in the soluble fractins, *i.e.* the glutathione reductase (acceptor added : oxidized glutathione, Fig. 1a) and the TPNH-diaphorase (acceptor added : 2 : 6-dichlorophenol indophenol, Fig. 1b).

B. *Ribolysis, formation of sedoheptulose and hexose**

(*a*) *Reaction 3† :* R5-P → Ru5-P (pentose isomerase)
*Reaction 4 :* R5-P + Ru5-P → S7-P + T3-P (transketolase)
*Reaction 5 :* S7-P + T3-P → F6-P + tetrose-phosphate (transaldolase)
*Reaction 6 :* Ru5-P + tetrosephosphate → F6-P + T3-P (transketolase)
*Reaction 7 :* 2 T3-P → FDP (aldolase)

Incubation of the soluble tumour fractions with R5-P at 37°C led to the disappearance of pentose with concommitant formation of sedoheptulose, hexose and triose. These findings can be covered by the reaction sequence described in reactions 3-7. Any or all of the three different ways of hexose formation may be followed (reaction 5, 6 and 7).

* For a review of the reactions taking place in the extra-glycolytic breakdown of glucose compare Racker (1954), Dickens (1956) and Horecker and Mehler (1955).

† Recent investigations (Srere *et al.*, 1955) have revealed that still another reaction may be involved, e.g. the epimerization of Ru5-P to xylulose 5-phosphate (X5-P) by a phosphoketopentose isomerase. In that case Ru5-P of reaction 4 and 6 has to be replaced by X5-P.

Pentose disappearance was followed using the orcinol reaction; Fig. 2, illustrating an experiment with the soluble fraction of the transplanted mouse sarcoma UV 256, shows that concommitantly with the decrease in optical density at 660 mμ, the slopes of the individual curves between 660 and 550 mμ. changed. This change is not only due to the disappearance of pentose, but also to the formation of sedoheptulose, which according to Horecker, Smyrniotis and Klenow (1953) displays a very weak band at 580–600 mμ. in the orcinol reaction.

FIG. 1*a*.—Coupled oxidation-reduction reaction between glucose 6-phosphate, triphosphopyridine nucleotide and glutathione by the soluble fractions of two transplanted mouse tumours.

T 5441 = granulosa cell tumour of the ovary : ▲——▲.
T 24202 = sarcomatoid ovarian tumour : ○——○.

1·0 ml. 0·25 M *Tris* (pH 7·6), 1·0 ml. 0·1 M $MgCl_2$, 0·2 ml. 0·05 M, G6-P, 1·0 ml. 0·0004 M glutathione (GSSG), 400 μg. TPN in 0·4 ml. water, 0·4 ml. enzyme preparation (dialyzed supernatant from a 10 per cent homogenate). Incubation at 37°C. At various times 1 ml. aliquots were transferred to 3·0 ml. 3 per cent metaphosphoric acid. The resulting mixture was saturated with 1·5 g. NaCl and centrifuged, in 2·0 ml. aliquots of the clear solution. GSH was assayed according to Grunert and Philips (1951).

Since the absorption spectra of glucose, fructose and triose do not show any difference in optical density between 660 and 600 mμ. (Newburgh and Cheldelin, 1955) pentose concentration is directly proportional to the difference in optical densities at these two wave lengths provided that a correction for sedoheptulose is made. The quantitative determination of heptulose was accomplished with the aid of the cysteine-sulfuric acid procedure (compare experimental part). Fig. 3a and 3b serve to illustrate two experiments in which R5-P was found to be converted to heptulose (510 mμ.) and to hexose (420 mμ.) by the soluble preparations from the sarcoma UV 256 and the ovarian tumour (granulosa cell type) T 5441, respectively.

Quantitative data on the disappearance of R5-P and the formation of S7-P are given in Fig. 4 and 5. A complete analysis of the formation of hexose, sedoheptulose and triose from R5-P after successive times of incubation is presented in Table II. Triose phosphate was measured as alkali-labile phosphate and hexose by the use of the anthrone colorimetric method, corrected for heptulose

FIG. 1b.—Coupled oxidation-reduction reactions between glucose 6-phosphate, triphosphopyridine nucleotide, and 2 : 6-dichlorophenol indophenol by the soluble fractions of some transplanted mouse tumours.

T 5441 = granulosa cell tumour : ▲——▲.
T 24202 = sarcomatoid ovarian tumour : ○——○.
T 5358 = interstitial cell carcinoma of the testis : □——□.

0·5 ml. 0·25 M *Tris* (pH 7·6), 0·5 ml. 0·25 M $MgCl_2$, 0·1 ml. 0·05 M G6-P, 200 μg. TPN, 25 μg. 2 : 6-dichlorophenol indophenol (DIP) and 0·1 ml. enzyme preparation in a total volume of 2·5 ml. Incubation at 20°C. DIP reduction was read at 610 mμ.

and triose. Results obtained with the paper chromatographic methods, mentioned in the experimental part, confirmed the spectrophotometric data concerning the presence of the various sugar phosphates.

(*b*) Since the various enzymes of the HMP oxidative pathway appeared to be present in the soluble tumour fractions, it should be possible to show in one experiment the synthesis of G6-P from R5-P (reactions 3–6 ; F6-P of reaction 5 or 6 can be converted into G6-P by the action of phosphohexoisomerase, which is also present) followed by an oxidation of G6-P on addition of TPN (reactions 1 and 2).

FIG. 2.—Disappearance of ribose 5-phosphate after incubation with the soluble fraction of the mouse sarcoma UV 256, as measured by the orcinol reaction.

The incubation mixture consisted of 0·5 ml. 0·05 M R5-P, 1·0 ml. 0·25 m *Tris* (pH 7·6), 1 ml. 0·1 M $MgCl_2$, 0·4 ml. enzyme preparation ; total volume 5·0 ml. Incubation at 37°C in air. At the times indicated (Curves 1–6 ; 0, 20, 40, 60, 120 and 180 minutes) 0·5 ml. of the incubation mixture was transferred to 4·5 ml. 5 per cent TCA, 0·3 ml. of the resulting deproteinized solution was used for the orcinol reaction.

FIG. 3*a*.—Conversion of ribose 5-phosphate to hexose and heptulose by the soluble fraction of the mouse sarcoma UV 256, as measured by the cysteine-sulfuric acid method.

For experimental conditions compare Fig. 2.

Curves 1–8 illustrated the spectra obtained from aliquots (one ml. of the deproteinized solutions was used for the cysteine-sulfuric acid test), taken at 10, 30, 60, 90, 120, 180, 300 and 360 minutes respectively.

FIG. 3*b*.—Conversion of ribose 5-phosphate to hexose and heptulose by the soluble fraction of the granulosa cell tumour of the ovary T 5441, as measured by the cysteine-sulfuric acid method.

Experimental conditions similar to those of Fig. 2, except that 0·2 ml. 0·05 M R5-P was used. Curves 1–7 illustrate the spectra obtained from aliquots taken at 0, 30, 60, 120, 180, 240 and 480 minutes respectively.

FIG. 4.—Breakdown of ribose 5-phosphate and formation of sedoheptulose by soluble preparations from normal and tumour tissues (long-term experiments).

For experimental conditions compare Fig. 2. Appropriate aliquots of the deproteinized solution were assayed for pentose and heptose.

●——● Rat heart, ○——○ mouse sarcoma UV 256, +——+ mouse hepatoma T 28012, ▲——▲ mouse liver, □——□ mouse lymphocarcoma T 86157.

TABLE II.—*Formation of Heptose, Hexose and Triose from Ribose 5-phosphate by the Soluble Fraction of the Mouse Ovarian Tumour T 5441 (granulosa cell type).*

Tubes containing 0·4 ml. 0·25 M *Tris* (pH 7·6), 0·4 ml. 0·1 M $MgCl_2$, 0·2 ml. 0·05 M R5-P, 0·16 ml. enzyme preparation in a total volume of 2·0 ml. were incubated at 37°C in air for the various times indicated. Reaction was stopped by adding 1·6 ml. of 10 per cent TCA. 2·0, 0·18, 0·05 and 0·18 of the deproteinized reaction mixtures were used for the determination of triose phosphate, the cysteine-sulfuric acid-, the orcinol- and the anthrone reaction, respectively. The blanks contained all components except R5-P.

| | μ-Mole, after (minutes) | | | | | |
|---|---|---|---|---|---|---|
| Compound. | 0. | 30. | 60. | 90. | 120. | 180. |
| Pentose . . | 10 | 7·1 | 6·3 | 4·8 | 4·5 | 3·7 |
| Heptose . . | 0 | ·32 | ·58 | ·82 | ·88 | ·92 |
| Hexose . . . | 0 | ·20 | ·40 | 1·0 | 1·6 | 2·2 |
| Triose . . . | 0 | ·60 | ·67 | ·76 | ·85 | ·78 |

This was accomplished in the following way: At 60, 120 and 180 minutes after incubation of the soluble fraction of the ovarian tumour T 5441 with R5-P, two 0·5 ml. aliquots were removed each time. To one tube of each pair of samples 200 μg. TPN in 0·1 ml. water was added, the other received water only. Subsequently the tubes were left at room temperature for half an hour before the reaction was stopped by adding TCA. As might be expected, the hexose concentration in each of the three reaction mixtures fortified with TPN was

FIG. 5.—Formation of sedoheptulose from ribose 5-phosphate by soluble preparations from mouse tumours (short-term experiments).

For experimental conditions compare Fig. 2.

△——△ luteoma T 7331, +——+ lymphosarcoma T 86157, ▲——▲ *ibid.*, □——□ mammary carcinoma T 8013, ●——● sarcoma UV 256, ○——○ *ibid.*, ▽——▽ mammary carcinoma T 98695.

Pentose breakdown at 37° C in enzyme units per gram fresh tissue amounted to 667, 615, 497, and 531 for the first four tumours respectively.

lower than their respective controls (Fig. 6). Sedoheptulose concentration did not change significantly.

(*c*) Extracts incubated with G6-P and TPN did not form sedoheptulose at 20°C, but did so at 37°C. Table III illustrates the disappearance of hexose and the formation of heptose (reactions 1–2 ; 3-keto 6-PG → Ru5-P + $CO_2$ ; 3–4) for a typical experiment.

The possibility that part of the sedoheptulose would be an artefact arising from the breakdown products of TPN was excluded by the finding that no ribose nor heptulose could be detected by paperchromatographic or spectrophotometric means following incubation of the tumour extracts with TPN at 37°C.

(*d*) *Reaction 8 :* G6-P → F6-P (phosphohexose isomerase).

When the tumour extracts were incubated in the presence of G6-P while TPN was omitted, a rise in optical density at 420 mμ. was noted after the cysteine-sulfuric acid method was applied to aliquots of the reaction mixture at successive times. This pointed to the formation of F6-P since the molar extinction of fructose is about twice that of glucose at this wave length. Taking this into account the concentration of glucose and fructose were calculated after it was found that no other compounds had been formed (Table IV).

(*e*) The formation of sedoheptulose could also be demonstrated by incubating the tumour extracts with G6-P and FDP. For example, 0·30 ml. of a dialysed

FIG. 6.—Hexose formation from ribose 5-phosphate by the soluble preparation of the ovarian tumour T 5441 both in the presence and absence of triphosphopyridine nucleotide.

The original incubation mixture consisted of 0·6 ml. 0·05 M R5-P, 1·2 ml. 0·25 M T*ris* (pH 7·6), 1·2 ml. 0·1 M $MgCl_2$ and 0·48 ml. enzyme preparation ; total volume 6·0 ml. After 1, 2 and 3 hours twice 0·5 ml. was transferred and incubated with 0·1 ml. of the fresh soluble enzyme preparation and either 0·1 ml. water containing 200 μg. TPN or 0·1 ml. water alone. Incubation for 30 minutes at room temperature. After this time 4·3 ml. 5 per cent TCA was added and 1·0 ml. of the deproteinized solution was used for the cysteine-sulfuric acid test. The resulting spectra are illustrated ; solid lines : from aliquots which had been incubated in the presence of TPN, broken lines : from aliquots which had been incubated in the absence of TPN.

TABLE III.—*Formation of Sedoheptulose from Glucose 6-phosphate by the Soluble Fraction of the Mouse Ovarian Tumour 5441 (Granulosa Cell Type), Incubated in the Presence of TPN.*

The reaction mixture consisted of 0·42 ml. 0·25 M T*ris*, 0·3 ml. 0·1 M $MgCl_2$, 0·15 ml. 0·05 M G6-P, 0·12 ml. enzyme preparation and 0·5 ml. 0·1 M NaOH to bring the reaction mixture, which contained also 11 mg. TPN, to a pH of 7·4. Incubation at 37°C in air. At successive times 0·2 ml. of the reaction mixture was pipetted into 1·8 ml. of 5 per cent TCA. 1·0 ml. and 0·3 ml. were used for the cysteine-sulfuric acid and the anthrone method, respectively. Simultaneously a reaction mixture containing all components except G6-P was run during the same period. Blanks containing water were treated as the test solutions.

| | μ-Mole, after (minutes) | | | | |
|---|---|---|---|---|---|
| Compound. | 0. | 15. | 30. | 60. | 120. |
| Hexose . . | 6·0 | 4·4 | 2·8 | 0·92 | 1·3 |
| Sedoheptulose . | 0 | ·08 | ·10 | ·38 | ·53 |

supernatant prepared from a 10 per cent homogenate of the luteoma T 7331, produced 0·70 $\mu$-mole sedoheptulose from G6-P and FDP in 60 minutes. In this system the substrate for sedoheptulose formation is furnished by the transketolase-catalyzed reaction between F6-P and glyceraldehyde 3-P (reverse of reaction 6). F6-P is formed by reaction 8 catalyzed by phosphohexoisomerase, and glyceraldehyde 3-P by the aldolase catalyzed-reaction 7. The pentose phosphate produced will yield S7-P by reaction 3 and 4, but another route is provided by the aldolase catalyzed reaction between tetrose-P and dihydroxyacetone phosphate, recently described by Smyrniotis and Horecker (1956), in which sedoheptulose 1, 7-diphosphate is formed or by reaction 5 using tetrose-P and F 6-P.

TABLE IV.—*Formation of Fructose from Glucose 6-phosphate by the Soluble Fraction of the Mouse Sarcoma UV 256.*

This experiment was performed exactly in the same way as that illustrated in Table III, except for the fact that TPN was omitted. The conversion of glucose into fructose was calculated taking the ratio 2 : 1 in the molar extinction coefficients of fructose and glucose (cysteine-sulfuric acid method) into account.

| | $\mu$-Mole, after (minutes) | | | |
|---|---|---|---|---|
| Compound. | 0. | 60. | 120. | 150. |
| Glucose . . | 6 | 2·8 | 3·5 | 2·5 |
| Fructose . . | 0 | 3·2 | 2·5 | 3·5 |

### II. *Experiments with slices*

### A. *Formation of sedoheptulose from endogenous sources*

First, the concentration and the rate of dissimilation of endogenous hexose was studied. Slices of the fresh tumours were cut and incubated in a Krebs Ringer phosphate buffer of pH 7·4 at 37°C with shaking in air. The two hepatomas of the mouse, T 26473 and T 28012, contained a relatively high concentration of hexose-positive reacting material in the anthrone test which fell to less than half of the original value on incubation during two hours. No convincing evidence for sedoheptulose formation was found. The hexose content of the other tumours amounted to 2–3 $\mu$-mole per gram fresh weight at the most ; during a two hour incubation period 0–50 per cent of the hexose disappeared. A slight formation of sedoheptulose was seen after this period in slices of the ovarian tumour T 5441, the testicular tumour T 5358 and the lymphosarcoma T 86157 only. In fresh tumour tissue no heptulose could be detected by the procedures employed. The amount of heptulose produced from the endogenous substrate by the tumours just mentioned never exceeded more than ten per cent of that formed from added G6-P (compare Section II, B).

### B. *Formation of sedoheptulose from added glucose and glucose 6-phosphate*

The concentration of glucose, which was added to the slices (7·5 $\mu$-mole per 500 mg. wet weight of tissue) fell off rapidly on incubation (Table V). At no time however, did sedoheptulose appear to be present ; not even in those cases in which the heptose had been found to be formed from endogenous substrate.

TABLE V.—*Formation of Sedoheptulose from Glucose and Glucose 6-phosphate by Slices of the Sarcomatoid Ovarian Tumour T 24202.*

500 mg. slices wet weight; 7·5 $\mu$-moles glucose or G6-P in a volume of 2·5 ml. Krebs Ringer phosphate buffer of pH 7·4. Incubation in air at 37°C. The concentrations of hexose listed in the table are those measured for the sum of endogenous and added hexose (compare text, Section II, A).

| Compound added. | | $\mu$-Mole, after (minutes). 60. | 120. |
|---|---|---|---|
| Glucose | Hexose | 2·5 | 1·1 |
| | Sedoheptulose | 0 | 0 |
| G6-P | Hexose | 7·0 | 6·5 |
| | Sedoheptulose | ·45 | ·60 |

In marked contrast with the results following addition of glucose are those obtained with G6-P. Although the concentration of the latter compound fell off rather slowly, as revealed by the anthrone test, a significant rise in the optical density at 510 m$\mu$. using the cysteine-sulfuric acid method* was found, which indicated the formation of sedoheptulose (Table V).

All the tumours which have been studied behaved exactly similarly: no sedoheptulose was formed from glucose, but the heptose was found to be present after incubation with G6-P both under aerobic and anaerobic conditions.

C. *Formation of sedoheptulose from added ribose 5-phosphate*

Incubation of slices from the ovarian tumours (sarcomatoid and granulosa cell type) with R5-P for various times yielded sedoheptulose and significant quantities of hexose (Table VI). In a few experiments in which the gas phase consisted of a 95 per cent $N_2$ : 5 per cent $CO_2$ mixture, the yield of sedoheptulose and hexose synthesized from R5-P was equal to that obtained under aerobic conditions (air). No formation of hexose could be observed when ribose was incubated with the slices.

TABLE VI.—*Formation of Sedoheptulose and Hexose from Ribose 5-phosphate by Slices of Two Ovarian Tumours (T 24202 and T 5441).*

500 mg. slices wet weight; 7·5 $\mu$-mole R5-P in 2·5 ml. Krebs Ringer phosphate buffer of pH 7·4. Incubation in air 37° C.

| Tumour. | Compound. | $\mu$-Mole, after (minutes) 0. | 30. | 60. | 120. | 180. |
|---|---|---|---|---|---|---|
| T 24202 | Sedoheptulose | 0 | — | ·34 | ·50 | — |
| | Hexose | 1·0 | — | 5·0 | 5·0 | — |
| T 24202 | Sedoheptulose | 0 | ·70 | ·68 | ·42 | ·42 |
| T 5441 | Sedoheptulose | 0 | — | ·48 | ·46 | — |
| | Hexose | 1·0 | — | 3·8 | 3·7 | — |

* The hexose maximum at 420 m$\mu$. may even be higher than at the start of the experiment, indicating the conversion of G6-P into F6-P.

D. *Effect of monoiodoacetate on the accumulation of intermediates*

Incubation of glucose together with MIA ($5 \cdot 4 \times 10^{-4}$ M) resulted in a marked retardation of glucose dissimilation with accumulation of F6-P (compare Section I, B, d) and of triose†, but, again, no sedoheptulose could be detected. When G6-P served as substrate, sedoheptulose formation in the presence of MIA was equal or slightly higher than in its absence.

After incubation of tumour slices with R5-P and MIA, the amount of sedoheptulose formed was not different from that found when the incubation had been carried out in the absence of MIA.

### III. *Experiments with Homogenates*

Homogenates were prepared and incubated in an atmosphere of 95 per cent $N_2$ : 5 per cent $CO_2$ according to the procedures described by Novikoff, Potter and Le Page (1948) The tumours T 5441 and T 86157 were studied first. 0·3 ml. aliquots of the homogenates (10 per cent in isotonic KCl-buffer) were incubated with G6-P (15 $\mu$-mole) in the presence of nicotinamide, fluoride, K-pyruvate and ATP, in a total volume of 3·0-ml. In the complete reaction mixture with DPN and TPN present, sedoheptulose was formed. When DPN, or DPN and TPN, were omitted the formation of sedoheptulose was not diminished. On the contrary, under the latter conditions the greatest amount of sedoheptulose was found, while the hexose disappearance was minimal as compared with the other experiments (Table VII).

TABLE VII.—*Formation of Sedoheptulose from Glucose 6-phosphate by a Fortified Homogenate of the Lymphosarcoma T 86157.*

The reaction mixture consisted of 0·3 ml. 0·1 M $(NH_4)_3PO_4$, 0·3 ml. 0·5 M KCl in 0·5 M $KHCO_3$, 0·2 ml. 0·01 M K-ATP, 0·3 ml. 0·4 M nicotinamide, 0·1 ml. 0·1 M $MgCl_2$, 0·1 ml. 0·15 M K-pyruvate, 0·19 ml. 0·16 M NaF, 0·3 ml. 0·05 M G6-P.

0·3 ml. 10 per cent tumour homogenate in isotonic KCl (containing bicarbonate) (final pH 7·7–8·1). Added 1·5 mg. TPN plus 0·5 mg. DPN, 1·0 mg. DPN, 1·5 mg. TPN or water only. Total volume 3·0 ml. Incubation at 37° C in an atmosphere of 95 cent $N_2$ plus 5 per cent $CO_2$ for 1 or 2 hours.

| Coenzymes added. | Hexose disappeared after (minutes). 60. (μ-mole.) | 120. | Sedoheptulose formed after (minutes). 60. (μ-mole.) | 120. |
|---|---|---|---|---|
| DPN + TPN | 11·1 | 11·3 | ·28 | ·25 |
| DPN | 9·6 | 9·4 | ·44 | ·39 |
| TPN | 6·8 | 9·4 | ·54 | ·51 |
| None | 3·0 | 3·0 | ·81 | ·90 |

In the absence of DPN, TPN, ATP, nicotinamide (the inhibitor of the pyridine nucleotide-destroying enzymes), fluoride (the inhibitor of the "ATP-splitting

† The formation of triose was evident from the rise in optical density at 450 mμ (anthrone test). In the following experiments using MIA together with G6-P or R5-P, triose was also found to accumulate.

enzyme ") and pyruvate, sedoheptulose was still formed. For example, in an experiment with T 5441 0·65 and 0·98 $\mu$-mole sedoheptulose were found after 1 and 2 hours, respectively. Similar results were obtained with a number of other tumours.

In a number of experiments attempts were made to destroy the TPN, ATP and DPN which might still be present in the homogenate. This was accomplished by pre-incubation of the latter for 15 minutes at 37°C in the presence of sucrose mitochondria prepared according to Schneider and Hogeboom (1950) from a transplanted liver carcinoma of the rat or from the sarcomatoid ovarian tumour T 24202. The mitochondria from these tumours had been found to possess high TPN-, DPN- and ATP-splitting activities. (Emmelot, Bos and Brombacher, 1956).

To serve this purpose the mitochondria were freed from sucrose by washing with and dialysis against distilled water. Despite the pre-treatment of the homogenate with the mitochondria, sedoheptulose formation by the unfortified homogenate as described for the previous experiment, remained unchanged; hexose concentration did not fall.

In a separate experiment with the mitochondria incubated without the homogenate no heptose was found.

### DISCUSSION

The experiments with slices reported in the present communication have shown that sedoheptulose, an important intermediate of the extra-glycolytic pathway, could not be demonstrated when glucose was used as the substrate. Thus sedoheptulose did not accumulate although it was undoubtedly formed. However with G6–P, which is markedly slower metabolized than free glucose, probably as a result of a more rapid entrance of the latter into the cells, sedoheptulose was found to accumulate in the tumour slices. Sedoheptulose could also be demonstrated when fortified whole homogenates of the tumours were incubated with G6-P. Sedoheptulose formation was at its maximum and hexose disappearance markedly less when the two coenzymes of the glycolytic and extra-glycolytic pathway, DPN and TPN, were omitted from the incubation mixture. After pre-treatment of the homogenates with TPN-, DPN- and ATP-splitting enzymes from mitochondrial preparations of certain tumours, and subsequent incubation of the homogenate sedoheptulose was still formed in the absence of added coenzymes, inhibitors (fluoride and nicotinamide), pyruvate, and ATP. It is improbable that under the latter conditions heptose originated from ribose produced by the direct oxidative decarboxylation of G6-P. Sedoheptulose formation by the pathway described under Section I, B, e, *i.e.* from F6-P and T3-P, would be more plausible. The former compound can easily be formed from added G6-P, whereas the latter or its direct precursor FDP has to be present in the original homogenate to account for this reaction.

It appeared further, that sedoheptulose accumulation was favoured under conditions in which glycolysis was abolished or retarded. A similar inverse relation between substrate metabolism via the glycolytic path and sedoheptulose accumulation was found in the slice experiments using glucose and G6-P. However, in the presence of MIA, which blocked the glycolytic pathway, glucose did not give rise to sedoheptulose.

The experiments carried out with cell-free extracts unambiguously demonstrated that the enzymes of the HMP-oxidative pathway were present in all the tumours studied. Apart from those described, still other reactions may be involved. Phosphorylation of Ru5-P and a carboxylation of the ribulose diphosphate formed with subsequent fission of the resulting six carbon compound into two molecules phosphoglyceric acid was postulated by Bassham, Benson and Calvin (1950) to account for the early appearance of the latter compound during their photosynthetic studies. The pertinent reactions have recently been demonstrated by Weissbach, Horecker and Hurwitz (1956) and by Jakoby, Brummond and Ochoa (1956). In accordance with this reaction sequence Barron and co-workers (1955) have found $^{14}C$ exclusively located in the carboxyl group of the lactic acid which was isolated after anaerobic incubation of tissue slices with $H^{14}CO_3^-$. Apparently these reactions were also involved in the pentose metabolism of the tumours studied by us. It was found (van Vals, Bosch and Emmelot, unpublished) that the lactic acid isolated after aerobic incubation of glucose-1-$^{14}C$ with slices from the transplanted mammary carcinoma T 49985, the lymphosarcoma T 86157 and the sarcomatoid ovarian tumour T 24202, contained three to five times as much isotope in its carboxyl group than did the corresponding group of the lactic acid originating from glucose-6-$^{14}C$. Both carbon atoms 1 and 6 of glucose are converted to the methyl group of lactate, but since $^{14}CO_2$ with a higher specific activity is released from glucose-1-$^{14}C$ than from glucose-6-$^{14}C$ during incubation of the tumour slices (van Vals, Bosch and Emmelot, 1956) the pentose carboxylation reaction may account for our findings.

SUMMARY

1. By the use of cell-free extracts, homogenates and slices of transplanted tumours, enzymes, intermediates and reactions participating in the hexose monophosphate oxidative pathway were demonstrated.

2. In the cell-free extracts, dehydrogenation involving glucose 6-phosphate and 6-phosphogluconate, ribolysis and formation of sedoheptulose, hexose, and triose were studied. All enzymes involved in the HMP oxidative pathway were found present.

3. Slices incubated in the presence of glucose 6-phosphate produced sedoheptulose. When added glucose was the substrate, sedoheptulose did not accumulate. On incubation with ribose 5-phosphate, sedoheptulose and hexose were produced.

4. In fortified and unfortified whole homogenates, sedoheptulose was formed from glucose 6-phosphate.

The authors wish to thank Miss H. J. de Boer and Mr. R. P. van Hoeven, for technical assistance and Dr. N. K. Richtmeyer (U.S.A.) for kindly providing us with sedoheptulosan monohydrate.

### REFERENCES

ABRAHAM, S., CADY, P. and CHAIKOFF, I. L.—(1956) *Proc. Amer. Ass. Cancer Res.*, **2**, 89.

*Idem*, HILL, R. AND CHAIKOFF, I. L.—(1955) *Cancer Res.*, **15**, 177.

AGRANOFF, B. W., BRADY, R. O. AND COLODZIN, M.—(1954) *J. biol. Chem.*, **211**, 773.

ALBAUM, H. G. AND UMBREIT, W. W.—(1947) *Ibid.*, **167**, 369.

ANDERSON, D. G., STAFFORD, H. A., CONN, E. E. AND VENNESLAND, B.—(1952) *Plant Physiol.*, **27**, 675.
AXELROD, B., BANDURSKY, R. S., GREINER, C. M. AND JANG, R.—(1953) *J. biol Chem.*, **202**, 619.
BARRON, E. S. G., VILLAVICENCIO, M. AND KING, Jr., D. W.—(1955) *Arch. Biochem.*, **58**, 500.
BASSHAM, J. A., BENSON, A. A. AND CALVIN, M.—(1950) *J. biol Chem.*, **185**, 781.
DICKENS, F.—(1956) *Third int. Congr. Biochem.*, Brussels, 1955. (C. Liébecq, ed.) New York (Academic Press), p. 170.
DISCHE, Z.—(1953) *J. biol. Chem.*, **204**, 983.
*Idem*, SHETTLES, L. B. AND OSNOS, M.—(1949) *Arch. Biochem.*, **22**, 169.
EMMELOT, P., BOS, C. J. AND BROMBACHER, P. J.—(1956) *Brit. J. Cancer*, **10**, 188.
DE FLINES, J.—(1951) *Experientia*, **7**, 234.
GLOCK, G. E. AND MCLEAN, P.—(1953) *Biochem. J.*, **55**, 400.—(1954) *Ibid.*, **56**, 171.
GRUNERT, R. R. AND PHILIPS, P. H.—(1951) *Arch. Biochem.*, **30**, 217.
HANES, G. S. AND ISHERWOOD, F. A.—(1949) *Nature*, **164**, 1107.
HAUGE, J. G., KING, T. E. AND CHELDELIN, V. H.—(1955) *J. biol Chem.*, **214**, 1.
HORECKER, B. L. AND MEHLER, A. H.—(1955) *Ann. Rev. Biochem.*, **24**, 207.
*Idem*, SMYRNIOTIS, P. Z. AND KLENOW, H.—(1953) *J. biol. Chem.*, **205**, 661.
JAKOBY, W. B., BRUMMOND, D. O. AND OCHOA, S.—(1956) *Ibid.*, **218**, 811.
KINOSHITA, J. H. AND MASURAT, T.—(1954) *Arch. Biochem.*, **53**, 9.
KIT, S.—(1956) *Cancer Res.*, **16**, 70.
*Idem* AND GRAHAM, O. L.—(1956) *Ibid.*, **16**, 117.
KLEVSTRAND, R. AND NORDAL, A.—(1950) *Acta chem. scand.*, **4**, 1320.
MEJBAUM, W.—(1939) *Z. physiol. Chem.*, **258**, 117.
MORTIMER, D. C.—(1952) *Canad. J. Chem.*, **30**, 653.
NEWBURGH, R. W. AND CHELDELIN, V. H.—(1955) *J. biol. Chem.*, **214**, 37.
NOVIKOFF, A. B., POTTER, V. R. AND LE PAGE, G. A.—(1948) *Cancer Res.*, **8**, 203.
RACKER, E.—(1954) *Advanc. Enzymol.*, **15**, 141.
RALL, T. W. AND LEHNINGER, A. L.—(1952) *J. biol. Chem.*, **194**, 119.
SCHNEIDER, W. C. AND HOGEBOOM, G. H.—(1950) *Ibid.*, **183**, 123.
SEEGMILLER, J. E. AND HORECKER, B. L.—(1951) *Ibid.*, **192**, 175.
SRERE, P. A., COOPER, J. A., KLYBAS, V. AND RACKER, E.—(1955) *Arch. Biochem.* **59**, 535.
SEIFTER, S., DAYTON, S., NOVIC, B. AND MUNTWYLER, E. —(1950) *Ibid.*, **25**, 191.
SMYRNIOTIS, P. Z. AND HORECKER, B. L.—(1956) *J. biol Chem.*, **218**, 745.
UMBREIT, W. W.—(1947) ' Manometric techniques '. Minneapolis (Burgess), p. 194.
VAN VALS, G. H., BOSCH, L. AND EMMELOT, P.—(1956) *Brit. J. Cancer*, **10**, 792.
WEISSBACH, A., HORECKER, B. L. AND HURWITZ, J.—(1956) *J. biol Chem.*, **218**, 795.
WENNER, C. E., BLOCH-FRANKENTHAL, L. AND WEINHOUSE, S.—(1956) *Proc. Amer. Ass. Cancer Res.*, **2**, 156.
WILLIAMS, K. T. AND BEVENUE, A.—(1951) *Arch. Biochem.*, **34**, 225.